# Editorial: Putting the “Why” Back into Bone “Archytecture”

**DOI:** 10.3389/fendo.2016.00014

**Published:** 2016-02-11

**Authors:** Phil Salmon

**Affiliations:** ^1^Bruker-microCT, Kontich, Belgium

**Keywords:** bone architecture, morphometry, growth and development, mechanotransduction, nonlinear pattern formation, remodelling, coupling, bone biomaterials

**The Editorial on the Research Topic**

**Putting the “Why” Back into Bone “Archytecture”**

This topic asked a question – can we do more than merely describe bone architecture, or analyze its mechanical performance? Can we get insights into the pattern forming mechanisms that generate bone’s adaptive architecture? First, I should express gratitude on behalf of myself and co-editors Daniel Chappard and Andy Pitsillides, to the authors who contributed articles. Out of busy academic schedules, they made time to write papers, which provide interesting and novel answers to the above question, from a number of different angles. Also to the reviewers who gave of their time generously, and to the Frontiers staff for their helpful and patient input.

In order to clarify the meaning of this topic with its strange title (containing a deliberate mis-spelling of “architecture”), I contributed an article (Salmon) “Non-linear pattern formation in bone growth and architecture.” More of a stream of consciousness on chaos and pattern than a conventional introduction-methods-results-discussion paper, it threw out some ideas of how chaos-related non-linear pattern processes may be evident on bone growth and architecture. The paper by Chappard et al. (3D Porous Architecture of Stacks of ß-TCP Granules Compared with That of Trabecular Bone: A microCT, Vector Analysis, and Compression Study) looked at the 3D architecture of an osteogenic scaffold. In terms of the question of how bone remodeling cells behave spatiotemporally within the bone marrow space, this scenario of the bone scaffold could hardly be more appropriate.

The article by Harrison and Cooper (Modalities for Visualization of Cortical Bone Remodeling: The Past, Present, and Future) directly took up the challenge of moving from the “how” to the “why” in the 3D study of bone remodeling units. They provided a review of studies of osteonal remodeling in cortical bone, covering imaging methodologies, both *ex vivo* and *in vivo*, which can image these structures and the osteonal canal networks. The study by Wu et al. (Using Micro-CT Derived Bone Microarchitecture to Analyze Bone Stiffness – A Case Study on Osteoporosis Rat Bone) studied the link between trabecular bone’s 3D architecture (as measured by microCT) and its mechanical performance in mechanical tests or finite element analysis (FEA). Erben in his paper “Hypothesis: coupling between resorption and formation in cancellous bone remodeling is a mechanically controlled event,” addressed directly the difficult question of how bone remodels on a mechanistic level to achieve a target architecture, which is mechanically adapted to the loads and load directions it experiences.

The study by Acquaah et al. (Early trabecular development in human vertebrae: overproduction, constructive regression, and refinement) was based on very rare and valuable datasets of prenatal, neonatal, and infant human vertebral bones, from a 19th century anatomical collection. This allowed a unique study of the 4D trajectory of growth and bone architecture at this site from 3 months before birth to 2.5 years age, which could prove extremely useful in examining the interaction of genetically determined “baseline” trabecular architecture with an increasing mechanically adaptive component. This connects directly with the paper by Erben on how bone remodeling cells respond to these loads.

Developing further the theme of mechanical loading and skeletal architecture, the paper by Galea et al. “Quantification of alterations in cortical bone geometry using site specificity software in mouse models of aging and the responses to ovariectomy and altered loading,” came from the lab of Lance Lanyon who is arguably the founder of the *in vivo* study of skeletal biomechanics and load transduction. Over the last half century, Lanyon and his colleagues have pioneered techniques now used world-wide to measure accurately the strains actually experienced in the bones of animal models under controlled loads. One important insight that has come from the work of this group is that the architectural response of a bone to loading must be studied along the bone as a whole, not restricted to a single site such as the midshaft. Galea and his colleagues found that changes in a long bone, in response to loading, disuse, ovariectomy and aging, are very different at different locations, since the “goal” of the bone modeling response is to change its overall shape.

Non-linear pattern formation in bone originates ultimately from the nature of interactions within and between communities of bone remodeling cells – the osteoclasts and osteoblasts. We were fortunate to have a paper contributed by leading authorities on this subject, Sims and Martin. Their paper asked the critical question: “Coupling signals between the osteoclast and osteoblast: how are messages transmitted between these temporary visitors to the bone surface?” In an illuminating focused short review, a detailed picture is given of the osteoclast-to-osteoblast coupling critical to the emergent morphology of bone. It turns out that there is much more to this coupling than the identification of cytokine and signal-receptor links. Microanatomy of the marrow stroma and structures like the resorption “canopy,” which transiently lifts over the BMU site like a protective umbrella, also play a role. The review provides a rich source of possible topics for further research into this coupling. The paper’s figure 1 is a wonderfully clear annotated cartoon of the resorption to reversal to formation sequence which will likely find its way into many presentations and thesis introduction chapters where bone remodeling essentials are communicated.

The paper by Gao et al. in the group of Janet Henderson in Montreal addressed the bone pathology scoliosis in which an abnormality of bone growth leads to asymmetric curvature of the spinal column (“Micro CT analysis of spine architecture in a mouse model of scoliosis”). This pathology can only be described with reference to 3D topography – a spinal axis which twists in 3D departing from bilateral symmetry. MicroCT analysis of the spines of a genetic mouse model of scoliosis allowed accurate geometric quantification of this disease, providing an important new tool to shed light on its etiology at a mechanistic level. The paper by Doube “The ellipsoid factor for quantification of rods, plates, and intermediate forms in 3D geometries,” develops further the topic of quantitative morphometric measurement of bone architecture. Understanding of the complex architecture of especially trabecular bone requires the use of quantitative parameters to characterize important aspects of its architecture. Doube has proposed a new parameter, the ellipsoid factor (EF), which promises to assess plate-rod trabecular architecture, also providing stereological information critical to adaptive load bearing. This parameter is already challenging the existing paradigm of higher trabecular percent volume always meaning more plate-like structure.

In summary, then, the articles contributed were just what we hoped for a snapshot of leading edge bone biology research, which addresses the question of how bone gets its shape.

## Author Contributions

The author confirms being the sole contributor of this work and approved it for publication.

## Conflict of Interest Statement

Phil Salmon is an employee of Bruker-microCT.

